# A Single Polar Residue and Distinct Membrane Topologies Impact the Function of the Infectious Bronchitis Coronavirus E Protein

**DOI:** 10.1371/journal.ppat.1002674

**Published:** 2012-05-03

**Authors:** Travis R. Ruch, Carolyn E. Machamer

**Affiliations:** Department of Cell Biology, The Johns Hopkins University School of Medicine, Baltimore, Maryland, United States of America; Wadsworth Center, NYSDH, United States of America

## Abstract

The coronavirus E protein is a small membrane protein with a single predicted hydrophobic domain (HD), and has a poorly defined role in infection. The E protein is thought to promote virion assembly, which occurs in the Golgi region of infected cells. It has also been implicated in the release of infectious particles after budding. The E protein has ion channel activity in vitro, although a role for channel activity in infection has not been established. Furthermore, the membrane topology of the E protein is of considerable debate, and the protein may adopt more than one topology during infection. We previously showed that the HD of the infectious bronchitis virus (IBV) E protein is required for the efficient release of infectious virus, an activity that correlated with disruption of the secretory pathway. Here we report that a single residue within the hydrophobic domain, Thr16, is required for secretory pathway disruption. Substitutions of other residues for Thr16 were not tolerated. Mutations of Thr16 did not impact virus assembly as judged by virus-like particle production, suggesting that alteration of secretory pathway and assembly are independent activities. We also examined how the membrane topology of IBV E affected its function by generating mutant versions that adopted either a transmembrane or membrane hairpin topology. We found that a transmembrane topology was required for disrupting the secretory pathway, but was less efficient for virus-like particle production. The hairpin version of E was unable to disrupt the secretory pathway or produce particles. The findings reported here identify properties of the E protein that are important for its function, and provide insight into how the E protein may perform multiple roles during infection.

## Author Summary

Coronaviruses are enveloped viruses that bud and assemble intracellularly, and therefore must use the host secretory pathway for release. Coronavirus E is a small protein that contains a single predicted hydrophobic domain and is targeted to the Golgi region. The E protein has been implicated in the assembly of coronavirus particles, as well as in virus release after assembly. The mechanism of action is not understood, but may involve ion channel activity. The membrane topology of the E protein is also unclear, and the protein may adopt distinct topologies that have different functions. We previously showed that the E protein from the infectious bronchitis virus could disrupt the secretory pathway to the apparent advantage of the virus. Here we have mapped this activity to a single, essential residue within the hydrophobic domain. Additionally, we developed mutant versions of IBV E that adopt a single membrane topology, and showed that a transmembrane topology is required for disruption of the secretory pathway. Our results broaden the understanding of E protein function and will impact the development of antiviral strategies.

## Introduction

Coronaviruses (CoVs) are enveloped, positive strand RNA viruses that infect a variety of mammalian and avian species. In humans, CoVs are responsible for nearly 20% of common cold cases. CoVs can also lead to more serious disease as seen during the outbreak of the severe acute respiratory syndrome coronavirus (SARS-CoV) in 2003. To better prepare for the emergence of another highly pathogenic CoV it is important to increase our understanding of CoV biology.

The CoV virion consists of a helical nucleocapsid, made up of the CoV N protein and the genome, surrounded by a lipid envelope. Three structural proteins are embedded in the virion envelope. The CoV S protein is a type I transmembrane protein and is responsible for the attachment and fusion of the virion during entry. The CoV M protein has three transmembrane domains and drives the organization of the virion through its interactions with the other structural proteins [1]. The CoV E protein is small (76–108aa), is predicted to contain a single hydrophobic domain (HD), and is a minor component of the virion envelope. CoV E and CoV M drive the assembly of the virion [2]. CoV assembly occurs intracellularly at the endoplasmic reticulum-Golgi intermediate compartment (ERGIC) [3]. This results in fully assembled infectious particles within the lumen of the Golgi complex and downstream secretory organelles. Thus, virions must use the host secretory pathway in order to reach the plasma membrane and be released from infected cells.

In addition to its role in assembly, CoV E may have other functions during infection. Studies in planar lipid bilayers have shown that CoV E has ion channel activity [4], [5]. These studies also showed that the small molecule hexamethylene amiloride (HMA) inhibits the ion channel activity of mouse hepatitis virus (MHV) E and human coronavirus 229E (HCoV 229E) E. While there is no direct evidence that CoV E acts as an ion channel during infection, addition of HMA to either MHV or HCoV 229E infected cells inhibits viral replication, and mutations introduced into the HD of MHV E impair virus production suggesting that the putative ion channel activity may play a role during infection [5], [6]. If CoV E acts as an ion channel, it must form higher order structures because it contains only one predicted transmembrane domain. Indeed, structural and computational studies have suggested that CoV E forms a homo-pentamer in the membrane with a pore in the middle [7]–[9]. Understanding the role of a pentameric E ion channel is an important question in the field.

The membrane topology of CoV E is of considerable debate. CoV E has a short (∼10aa) hydrophilic N-terminus followed by a long hydrophobic domain (∼25aa) and a hydrophilic C-terminus. The N-terminus does not contain a canonical ER signal sequence [10]. The hydrophobic domain is unusually long for a protein targeted to the ERGIC/Golgi complex, but does not appear to be long enough to span the lipid bilayer twice [11]. These properties make it difficult to predict the topology based on the primary sequence. Complicating matters is the fact that multiple topologies have been reported in the literature for different CoV E proteins. Both IBV E and SARS-CoV E have been reported to exist as a type III transmembrane protein (N_exo_, C_cyto_) [12], [13]. Other investigators have reported the opposite topology for SARS-CoV E and transmissible gastroenteritis virus (TGEV) E (N_cyto_, C_exo_) [14], [15]. Yet another topology reported for CoV E is a membrane hairpin, where the hydrophobic domain bends into the cytoplasmic leaflet of the membrane with the N- and C-termini in the cytoplasm. The hairpin topology has been reported for MHV E and SARS-CoV E [15]–[17]. These discrepancies suggest that CoV E may adopt more than one membrane topology. If this is the case, CoV E may perform distinct functions depending on how it is inserted into the membrane. For example, a transmembrane version of CoV E could oligomerize and act as an ion channel, whereas a membrane hairpin could drive virion budding.

Since CoVs assemble intracellularly, their virions must pass through the host secretory pathway for egress. How or if the secretory pathway is modified in infected cells is not well understood, but may involve the E protein. A version of TGEV lacking the E protein was unable to produce infectious particles, but electron microscopy revealed that immature virions were present in secretory organelles of infected cells [18]. Alanine insertion scanning mutagenesis of the HD of MHV E produced mutant viruses that showed a defect in the release of infectious particles [6]. These results demonstrate that CoV E is important for virion trafficking, but did not identify the mechanism. It has long been appreciated that CoV infection drives a rearrangement of host cell membranes including the Golgi complex [19]. More recently it was shown that during CoV infection virions appear in large virion-containing vacuoles derived from Golgi/ERGIC membranes [20]. Recently we showed that the E protein of IBV promotes the release of infectious particles. We also observed that expression of IBV E results in the disruption of anterograde protein traffic and causes the Golgi complex to disassemble, and that all of these effects were dependent on the HD of IBV E [21]. This finding linked the efficient release of particles to the alteration of the host secretory pathway, and demonstrated that IBV E has a role during infection beyond assembly.

In the present study we set out to determine what properties of the HD of IBV E were important for disrupting the secretory pathway. We performed alanine scanning mutagenesis on the HD and identified a key residue required for disrupting the secretory pathway. We also addressed the role of topology in disrupting the secretory pathway by designing mutant versions of IBV E that adopted either a transmembrane or a membrane hairpin topology. This allowed, for the first time, functional analysis of the two specific forms.

## Results

### A Single Polar Uncharged Residue in IBV E is Necessary for Disrupting Protein Trafficking

**Figure 1:**
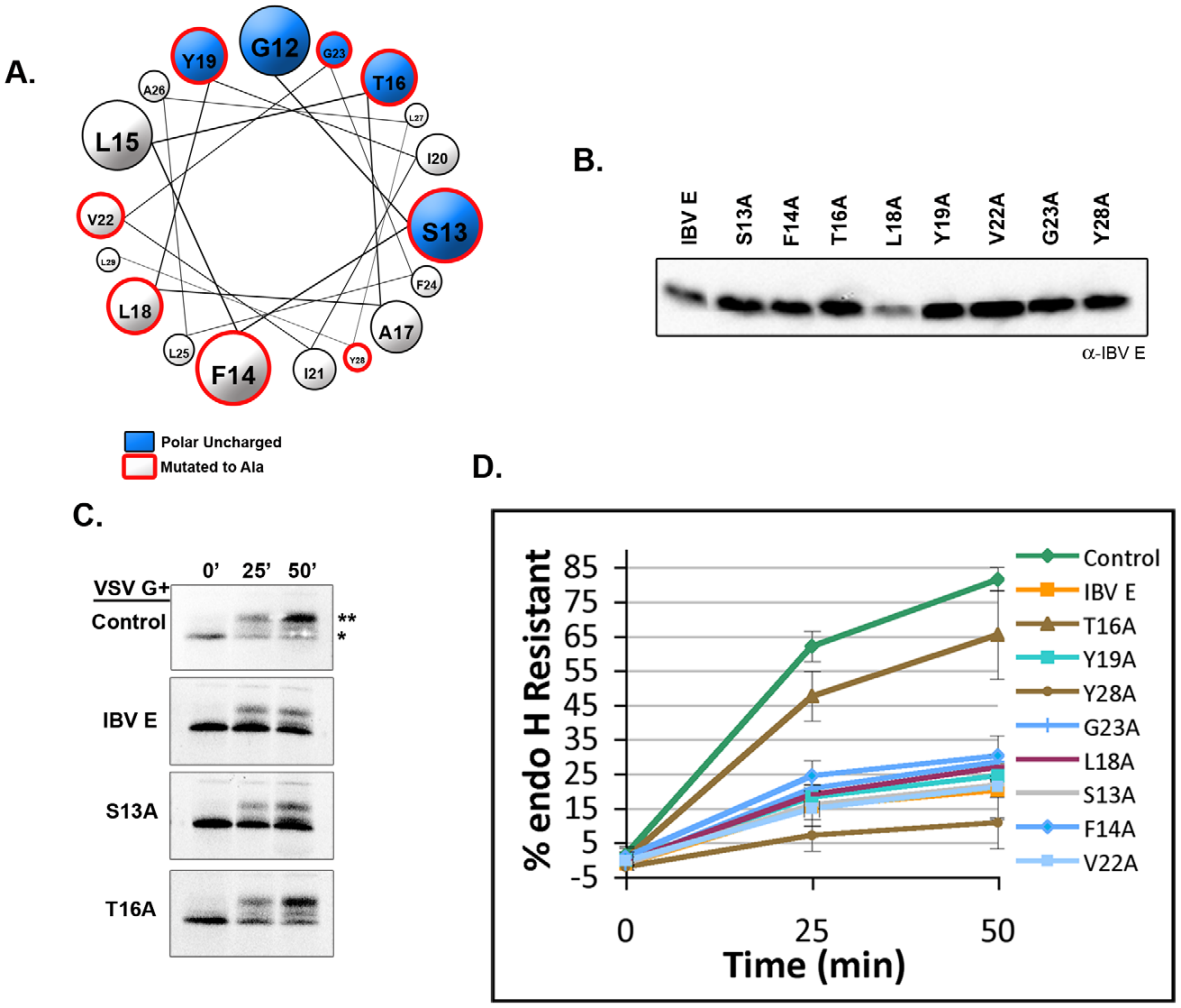
A single polar uncharged residue in IBV E is required for disruption of cargo trafficking. (A) A helical wheel diagram of the HD of IBV E. Polar uncharged residues are shown in blue; residues mutated to alanine are outlined in red. (B) An immunoblot shows that the alanine mutants of IBV E are expressed and run at a similar molecular weight when transiently expressed in HeLa cells. (C) VSV G was transiently co-expressed with the indicated protein in HeLa cells. 18–22 hours after transfection the cells were pulse-labeled with ^35^S-methionine/cysteine and chased for 0, 25, and 50 min. VSV G was immunoprecipitated from each sample and digested with endoglycosidase H. The mature (**) and immature (*) forms are indicated. Data from control, IBV E, S13A, and T16 A is shown. (D) Quantification of (C) showing that the T16A mutation inactivates the trafficking block. At each time-point the signal intensity for the mature and immature bands was measured. The percent of endo H resistant VSV G was calculated by dividing the signal for the mature band by the total signal (mature+immature). Data are from at least two independent experiments. Error bars represent +/− SEM.

When the HD of IBV E (GenBank ID: CAC39117) is modeled as an alpha helix and viewed in a helical wheel projection, polar uncharged amino acids cluster on one side (Figure 1A). If the cluster of polar uncharged residues is important for the disruption of the secretory pathway, mutating them to alanine should inhibit their effect, while mutations on the opposite side of the helix should have no effect. To test this hypothesis, single alanine mutations of the polar uncharged residues as well as residues on the opposite side of the helix were made. The mutant proteins were transiently expressed in HeLa cells and their expression was confirmed by immunoblot (Figure 1B). Next, we determined whether the mutants disrupted protein trafficking. The mutant proteins were expressed along with the model cargo protein vesicular stomatitis virus glycoprotein (VSV G). Trafficking of VSV G was measured using metabolic labeling in a pulse-chase assay coupled with endoglycosidase H (endo H) digestion. Since glycoproteins become resistant to digestion with endo H in the medial-Golgi, this assay monitors the rate at which a glycoprotein moves through the Golgi complex. All of the alanine mutants disrupted trafficking with the exception of IBV E T16A (Figure 1 C and D). Thus, a single polar uncharged residue within the HD of IBV E is necessary for disrupting protein trafficking.

### T16 Is Required for Disruption of the Golgi Complex

**Figure 2:**
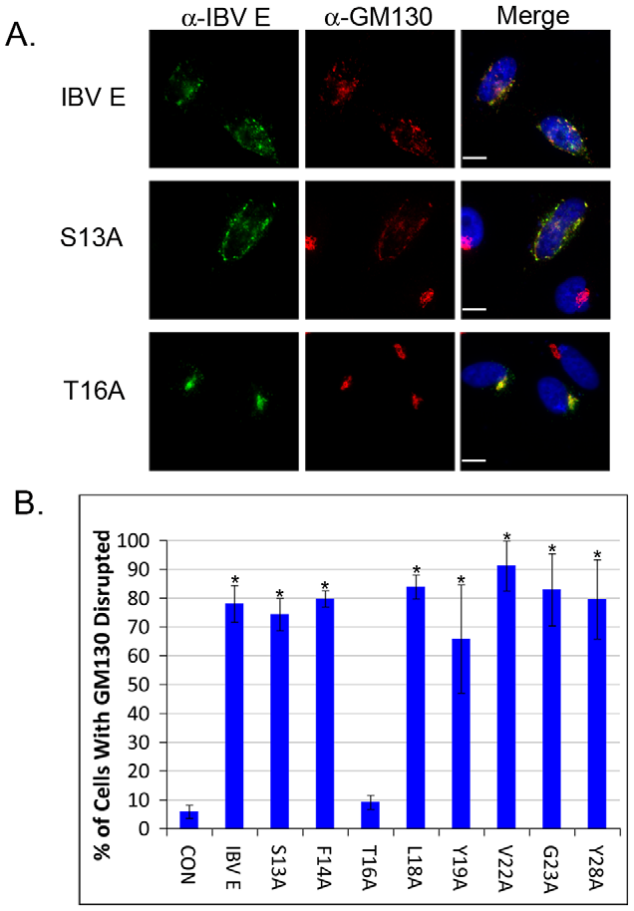
T16 is required for Golgi complex disruption. (A) Indirect immunofluorescence microscopy of HeLa cells transiently expressing IBV E, S13A, or T16A. IBV E is shown in green, GM130 is shown in red and nuclei are shown in blue. (B) Quantification showing the extent to which IBV E and the HD mutants disrupt Golgi complex morphology. To determine the extent of Golgi disruption, the area encompassing GM130 staining was measured in non-transfected cells and in cells expressing the various E mutants as described in Materials and Methods. Scale bars, 10 µm. Data are from three independent experiments, N≥54 for each condition. Error bars represent +/− SEM, and the asterisk denotes a significant increase in Golgi disruption compared to the control by Student's *t*-test (p≤5.4×10^−3^).

In addition to disrupting protein trafficking, IBV E expression disrupts Golgi morphology [21]. As with the trafficking defect, the disruption of the Golgi complex is dependent on the HD of IBV E. We reasoned that if the trafficking defect and Golgi complex disruption were occurring by the same process, T16 would be necessary for both effects. Indirect immunofluorescence microscopy was performed on cells transiently expressing IBV E or the mutant proteins. Cells were stained for IBV E and GM130, a marker of the Golgi complex. All of the mutant proteins disrupted the Golgi complex like IBV E with the exception of T16A, which had no effect on Golgi complex morphology (Figure 2A and B). These results, along with the data shown in Figure 1, demonstrate that a single polar uncharged residue within the HD of IBV E (T16) is necessary for the disruption of the secretory pathway.

### T16 Does Not Tolerate Substitution of Conserved Amino Acids

**Figure 3:**
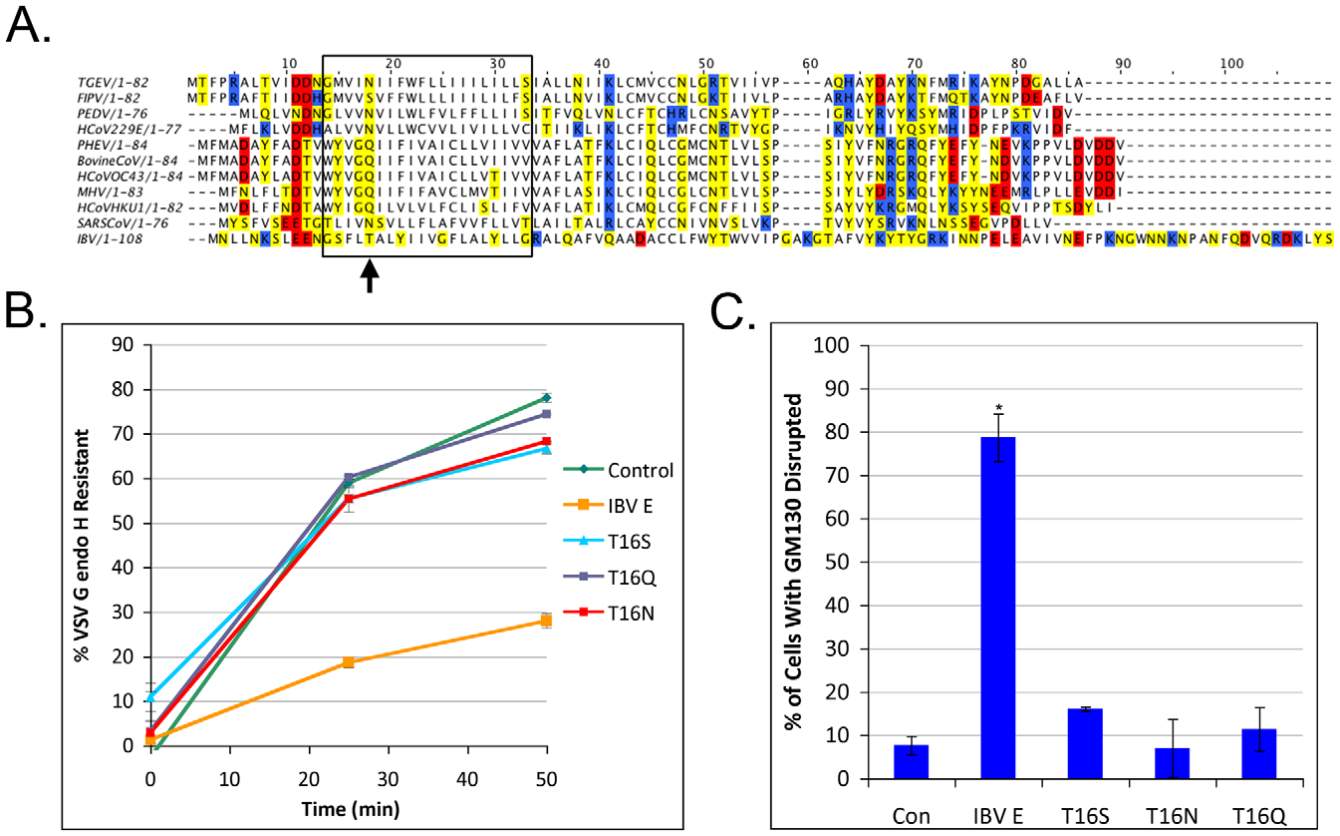
Substitution with other polar uncharged residues is not tolerated at position 16. (A) Multiple sequence alignment of CoV E proteins. Negatively charged residues are colored red, positively charged residues are colored in blue and polar uncharged residues are colored in yellow. The box encompasses the hydrophobic domain of IBV E, and the arrow denotes position 16 in IBV E. (B) VSV G pulse-chase coupled with endo H digestion as described in Figure 1. Mutation of T16 to S, N, or Q does not restore the ability of the protein to disrupt trafficking of VSV G through the Golgi complex. Data are from at least two independent experiments. Error bars represent +/− SEM. (C) IBV E protein with S, N or Q substituted for T16 does not induce Golgi complex disassembly (See Figure 2 for description of quantification). Data are from 3 independent experiments, N≥48 for each condition. Error bars represent +/− SEM, and the asterisk denotes a significant increase in Golgi disruption compared to the control by Student's *t*-test (p≤3×10^−5^).

We next determined if there was any flexibility in the amino acid required at position 16 in IBV E. A multiple sequence alignment of several different CoV E proteins showed that a polar uncharged residue is conserved at position 16 (Figure 3A). We introduced mutations at position 16 in IBV E that replaced the threonine with serine, asparagine or glutamine. The mutant proteins were transiently expressed along with VSV G to determine their effect on protein trafficking. None of the proteins disrupted trafficking of VSV G, showing that these residues could not substitute for threonine (Figure 3B). We examined the morphology of the Golgi complex in cells expressing the mutant E proteins using indirect immunofluorescence microscopy. Corroborating the trafficking results, none of the conserved mutations disrupted Golgi complex morphology as judged by GM130 staining (Figure 3C). Thus, there is a strict requirement for threonine at position 16 in IBV E.

### E Proteins with Mutations at T16 Support Virus-Like Particle Production

**Figure 4:**
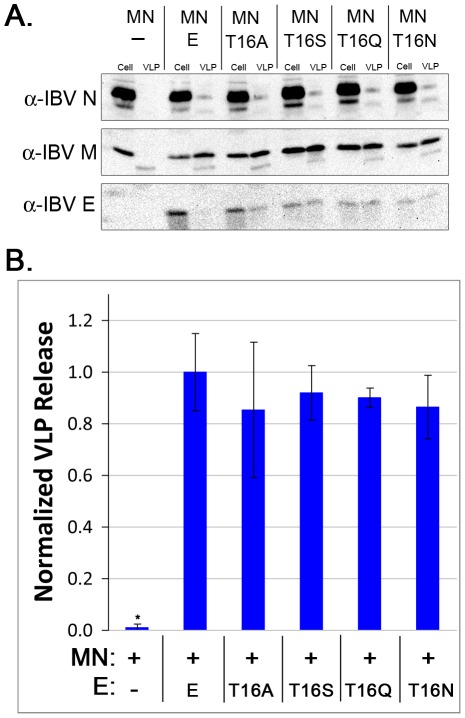
Mutations at T16 in IBV E support VLP production. (A) Immunoblot showing the amount of IBV N, M, and E in cells and released as VLPs. (10% of cell fraction, 100% of VLP fraction) (B) Quantification of immunoblot data showing the amount of M released with no E, IBV E, T16A, T16S, T16Q or T16N. Data were normalized to the amount of M expressed in each sample, and the amount of M released with IBV E was set to 1 for ease of comparison. Data are from three independent experiments. Error bars represent +/− SEM, and the asterisk denotes a significant decrease in VLP level compared to IBV E by Student's *t*-test (p<4×10^−3^).

Previously we reported that replacing the sequence of the HD of IBV E does not affect virus-like particle (VLP) production [22]. However, since these earlier experiments were carried out using a different cell type and expression system, we wanted to confirm that mutating T16 did not impair VLP production. We co-expressed IBV E and the T16 mutants along with plasmids encoding IBV M and IBV N in HeLa cells. The supernatant and cells were collected separately, and VLPs were purified from the supernatant via centrifugation over a sucrose cushion. The level of VLPs produced was measured by immunoblotting and comparing the signal for M in the VLP fraction to the cell fraction. We found that none of the mutations had a significant impact on steady-state VLP production as judged by the amount of M released (Figure 4A and B). Thus, T16 is required for altering the secretory pathway, but is not required for VLP production.

### Effect of Other CoV E Proteins on the Secretory Pathway

CoVs fall into three distinct groups based on genome similarities, alpha, beta and gamma. IBV is a gamma-CoV, and we wanted to determine if the effect on the secretory pathway was a property of other CoV E proteins. We transiently expressed the E proteins from the beta-CoVs SARS-CoV (GenBank ID: NP_828854.1) and MHV (GenBank ID: ACO72886) as well as the alpha-CoV TGEV (GenBank ID: ABG89321) in HeLa cells. Using antibodies directed against the various CoV E proteins or GM130, the morphology of the Golgi complex was examined. Somewhat surprisingly, none of the other CoV E proteins caused the Golgi complex to disassemble (Figure S1). Other markers for the Golgi complex were also distributed normally (data not shown). We determined if any of the other CoV E proteins impacted protein traffic through the Golgi complex using the pulse-chase endo H assay described above. VSV G trafficking was unaffected by expression of any of these E proteins (data not shown). Taken together these data indicate that the effect of SARS-CoV E, MHV E, and TGEV E on the host secretory pathway may be different than that of IBV E, and potentially point to an important difference in the function of the proteins. However, we found that the half-life of IBV E was longer (3.6 h) than that of MHV E (2 h), SARS-CoV E (2.1 h), or TGEV E (2.6 h) (data not shown). Additionally, we could not compare the absolute expression level of each protein (since the antibodies to detect each one are different). Thus, it is possible that MHV E, SARS-CoV E and TGEV E do not accumulate to as high a level as IBV E in this expression system, and therefore do not demonstrate the disruption in the secretory pathway observed for IBV E.

### Generation of IBV E Mutants That Adopt Either a Transmembrane or Membrane Hairpin Topology

**Figure 5:**
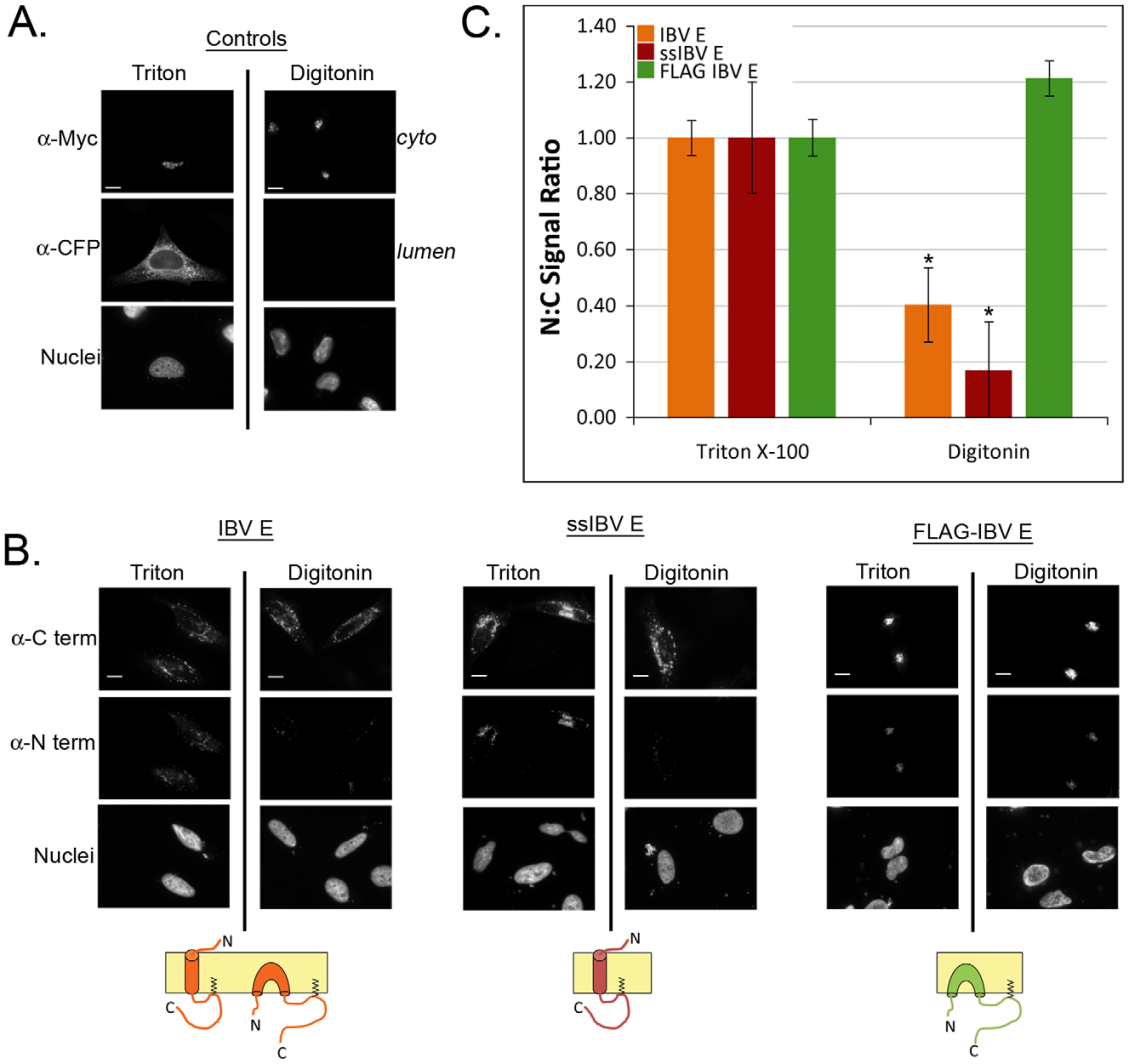
Generation of IBV E mutants that adopt distinct membrane topologies. (A) When cells are permeabilized with Triton X-100 both lumen (CFP-KDEL) and cytoplasmic (golgin160-Myc) epitopes are detected. Permeabilization with digitonin allows detection of the cytoplasmic epitope, but not the luminal epitope. (B) Selective permeabilization of cells expressing IBV E, ssIBV E, and FLAG-IBV E. The N-terminus of IBV E and ssIBV E was detected using a rabbit antibody to the N-terminus. The N-terminus of FLAG-IBV E was detected using a mouse anti-FLAG antibody. The C-terminus of each construct was detected using a rat-antibody against the C terminus of IBV E. Scale bars, 10 µm. Cartoons at the bottom of each panel show the predicted topology for each protein. (C) Quantification of topology shown as N-terminus to C-terminus fluorescence ratio (see Material and Methods). The data are normalized to the ratio from the Triton X-100 permeabilized samples. Data are from at least 2 independent experiments with N≥17 for each condition. Error bars represent +/− SEM, and the asterisk denotes a significant decrease in N∶C between the Triton X-100 and digitonin signal by Student's *t*-test (p≤3.4×10^−3^).

**Figure 6:**
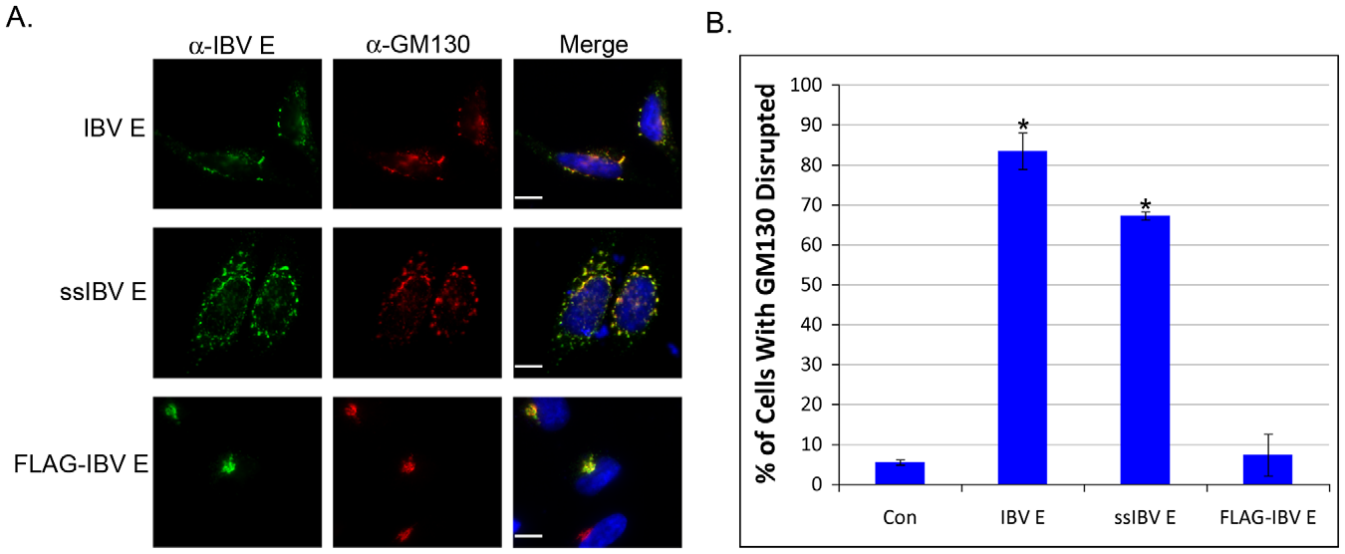
The transmembrane topology of IBV E promotes disruption of the Golgi complex. (A) Indirect immunofluorescence microscopy of HeLa cells transiently expressing IBV E, ssIBV E, or FLAG-IBV E. The E protein is shown in green, GM130 is shown in red, and nuclei are shown in blue. Scale bars, 10 µm. (B) Quantification of Golgi complex disruption in HeLa cells expressing IBV E, ssIBV E, or FLAG-IBV E (see Figure 2 for description of quantification). Data are from 3 independent experiments with N≥48 for each condition. Error bars represent +/−SEM, and the asterisk denotes a significant increase in Golgi disruption compared to the control by Student's *t*-test (p≤7.8×10^−6^).

Multiple groups have proposed different membrane topologies for the CoV E protein, either as a transmembrane protein or as a membrane hairpin (Figure 5B, cartoons) [10], [12]–[16]. It is possible that CoV E may adopt multiple membrane topologies, each with distinct function(s). To test the role of topology in IBV E function, mutant versions of IBV E were created with either a transmembrane or membrane hairpin topology. To promote a transmembrane topology we added a canonical cleavable N-terminal signal sequence onto the N-terminus of IBV E (ssIBV E), which will force the cleaved N-terminus into the ER lumen [23], [24]. To produce a potential membrane hairpin we added a FLAG tag onto the N-terminus (FLAG-IBVE). The rationale for this was that other N-terminally FLAG tagged CoV E proteins adopt a membrane hairpin topology [15], [16]. We transiently expressed IBV E, ssIBV E, and FLAG-IBV E in HeLa cells and probed their membrane topology using selective permeabilization of the plasma membrane with digitonin, followed by indirect immunofluorescence microscopy. As a control we co-expressed a luminal ER protein (CFP-KDEL) along with a protein present on the cytoplasmic side of the Golgi complex (golgin160-Myc). As expected, the cytoplasmic epitope of golgin160-Myc was accessible after either Triton X-100 or digitonin permeabilization, whereas the luminal epitope of CFP-KDEL was not accessible when cells were permeabilized with digitonin (Figure 5A). For IBV E and ssIBV E we stained for either the N- or C-terminus using antibodies directed to either end of the protein. We found that both IBV E and ssIBV E largely existed as transmembrane proteins with the N-terminus in the lumen and the C-terminus in the cytoplasm (Figure 5B). For FLAG-IBV E we used a similar approach but stained for the N-terminus with an anti-FLAG antibody because our anti-IBV E N-terminal antibody was unable to recognize the modified N-terminus. The results showed that FLAG-IBV E had both the N- and C-termini in the cytoplasm (Figure 5B). We also found that the mutations did not affect the targeting of either construct, as both colocalized with Golgi complex markers (Figure 6A and data not shown). We quantified the difference in the staining intensity under the different permeabilization conditions by measuring the fluorescence signal for the N- and C-termini in the same cell. After subtracting the background signal, the N∶C ratio was calculated, and normalized to the ratio from the Triton X-100 samples for ease of comparison (Figure 5C). As expected the ratio dropped dramatically for both IBV E and ssIBV E, but not for FLAG-IBV E. Thus, mutant IBV E proteins predominantly adopt either a transmembrane or membrane hairpin topology. It is worth noting that the N∶C ratio was lower for ssIBV E than for IBV E. While this difference was not statistically significant, we speculate that there may be a small population of IBV E that is inserted as a membrane hairpin.

### A Transmembrane Topology is Required for Disrupting the Secretory Pathway

Having developed versions of IBV E that adopt a unique orientation in the membrane, we determined if topology was important for disrupting the Golgi complex. IBV E, ssIBV E, and FLAG-IBV E were transiently expressed in HeLa cells and subjected to indirect immunofluorescence microscopy. Staining for IBV E and GM130 revealed that ssIBV E disrupted Golgi complex morphology to a similar degree as IBV E (Figure 6A and B). However, FLAG-IBV E had no effect on Golgi complex morphology (Figure 6A and B). This result suggests that the transmembrane topology is necessary for inducing Golgi complex disassembly. Since IBV E with mutations at T16 did not disrupt the secretory pathway, it was important to confirm that these mutations did not disrupt topology. Indeed, the selective permeabilization assay demonstrated that the topology of IBV E-T16A was identical to IBV E (Figure S2).

**Figure 7:**
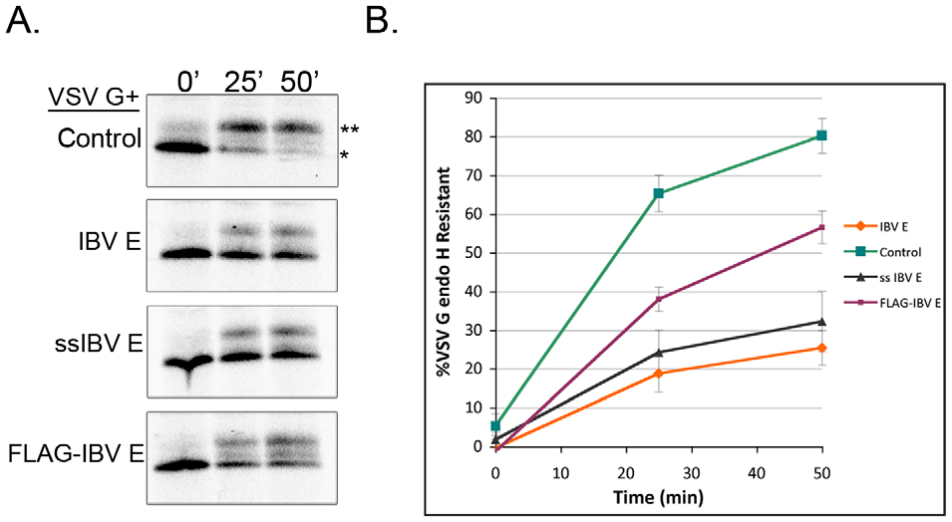
The transmembrane topology of IBV E promotes disruption of protein trafficking. (A) VSV G pulse-chase coupled with endo H digestion as described in Figure 1. The mature (**) and immature (*) forms are indicated. (B) Quantification of the pulse-chase data. Both ssIBV E and IBV E dramatically affect protein trafficking, while FLAG-IBV E has a more modest effect. Data are from 3 independent experiments. Error bars represent +/− SEM.

Next we tested whether expression of the topology constructs affected protein trafficking. We found that ssIBV E disrupted protein trafficking similar to IBV E (Figure 7A and B). FLAG-IBV E did not disrupt trafficking to the same extent as ssIBV E or IBV E but still had some effect (Figure 7A and B). This could indicate the IBV E hairpin does have some effect on trafficking, albeit to a smaller degree. It is also possible that a portion of FLAG-IBV E is inserted in a transmembrane topology and this small pool of protein is sufficient to alter trafficking, but insufficient to disrupt the morphology of the Golgi complex.

The addition of a FLAG tag to the N-terminus of IBV E could result in a number of effects beyond changing the topology of IBV E. Thus, we generated a version of IBV E that had a canonical signal sequence, followed by a FLAG tag on the N-terminus (ssFLAG-IBV E). When transiently expressed in HeLa cells, ssFLAG-IBV E was not targeted as well to the Golgi complex as the other constructs (Figure S3A). Also, selective permeabilization showed that a larger portion of the N-terminus was in the cytoplasm compared to ssIBV E (Figure S3B). These observations suggest that the FLAG tag may alter insertion and targeting when added behind a cleaved signal sequence. However, even with these caveats, ssFLAG-IBV E still disrupted trafficking similarly to IBV E and ssIBV E (Figure S3D). ssFLAG-IBV E also disrupted the Golgi complex, but to a lower degree than IBV E or ssIBV E, possibly due to less efficient targeting (Figure S3C). These results strongly support our interpretation of the importance of topology and IBV E function.

### Role of Membrane Topology in Assembly

**Figure 8:**
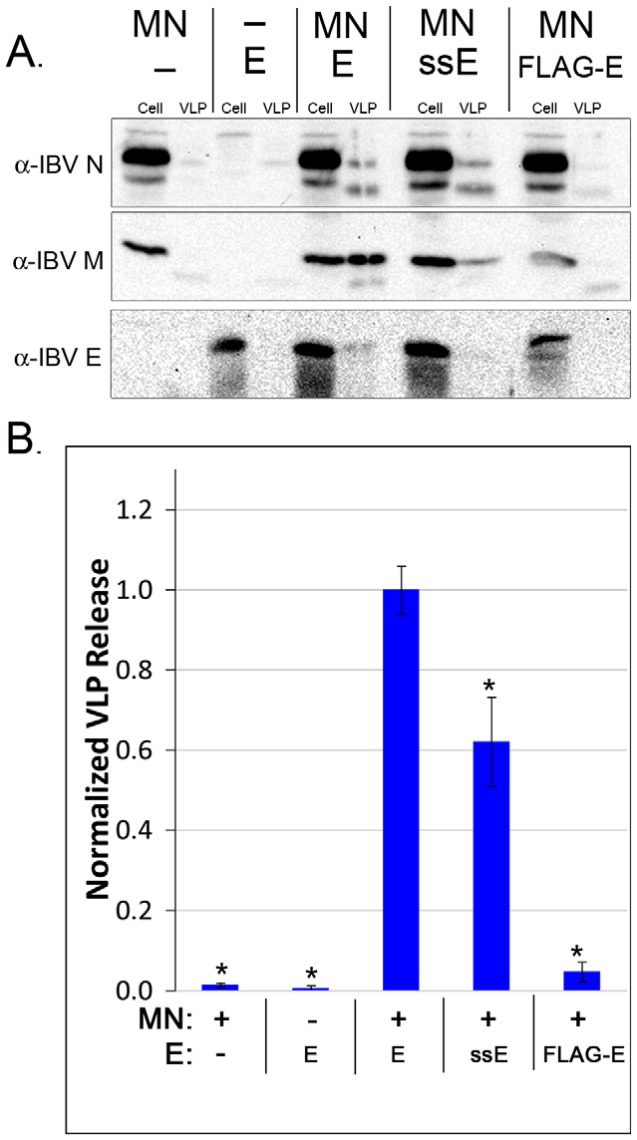
Neither ssIBV E or FLAG-IBV E produce normal levels of VLPs. (A) Immunoblot showing the amount of IBV N, M, and E in cells and released as VLPs. (10% of cell fraction, 100% of VLP fraction) (B) Quantification of immunoblot data showing the amount of M released with no E, IBV E, ssIBVE, or FLAG-IBV E. Data were normalized to the amount of M expressed in each sample, and the amount of M released with IBV E was set to 1 for ease of comparison. Data are from at least five independent experiments. Error bars represent +/− SEM, and the asterisk denotes a significant decrease in VLP level compared to IBV E by Student's *t*-test (p≤0.01).

To test how the membrane topology of IBV E affects particle assembly, we assayed IBV E, ssIBV E and FLAG-IBV E in a VLP assay. We co-expressed the E constructs along with plasmids encoding IBV M and IBV N in HeLa cells and determined the amount of VLPs released into the supernatant by immunoblotting (Figure 8A and B). Cells expressing ssIBV E produced less VLPs than those expressing wild-type IBV E, suggesting that the transmembrane topology can at least partially drive assembly, possibly by inducing membrane curvature in a lattice of IBV M. In support of this result, ssFLAG-IBV E also produced reduced levels of VLPs (Figure S3E). Cells expressing FLAG-IBV E produced almost no VLPs, indicating that the hairpin topology alone may not support the production of particles. This result is harder to interpret. It is not clear if the membrane hairpin is unable to drive assembly, release, or both.

## Discussion

We reported previously that replacing the entire HD of IBV E with a heterologous sequence eliminated disruption of the secretory pathway in transfected cells, and dramatically reduced the release of infectious virus from infected cells [21]. Total particle release was only modestly affected, however, suggesting that the HD of IBV E is important for preventing damage to virions during egress. Here we have shown that a single amino acid in the HD of IBV E (T16) is critical for disruption of the secretory pathway in cells expressing IBV E, but was not required for VLP production. This result suggests that the alteration to the secretory pathway is uncoupled from the role of E in assembly. Additionally, we generated versions of IBV E that adopted either a transmembrane or membrane hairpin topology. Using these mutants, we showed that a transmembrane topology was required for secretory pathway disruption. The residue equivalent to T16 in SARS-CoV E, N15, is predicted to lie in the pore region of a homo-pentamer [7]. Studies on a lysine-flanked peptide of the SARS-CoV E HD showed that N15 was important for the ion channel activity of the peptide in planar lipid bilayers [25]. Since we found that a transmembrane topology and T16 are required for disrupting the secretory pathway, and both are predicted to be important for ion channel activity, it is certainly possible that the disruption of the secretory pathway is due to the putative channel activity of IBV E. Alteration of Golgi complex structure and disruption of protein traffic occur when the ion balance at the Golgi complex is disrupted [26]–[29]. While an active ion channel at the Golgi complex could explain our observations, how altering the ion balance of secretory organelles might facilitate release of infectious particles remains unknown. We speculate that the demands of trafficking large virion cargo require the expansion of the Golgi complex cisternae, which may be achieved by changing the luminal ion concentration. Alternatively, a change in luminal environment may inactivate proteases present in the secretory pathway, thus protecting the virions from degradation that could render them non-infectious. The membrane rearrangements observed in CoV-infected cells are likely due at least partially to a disruption in the luminal microenvironment, although syncytia formation also contributes [19], [20]. Expression of the E protein in the absence of infection allowed us to assess its contribution to membrane rearrangements directly.

Many viruses encode small membrane proteins that have ion channel activity [30]. As a group these proteins are referred to as viroporins. The best studied viroporin is influenza M2, which forms a tetrameric pH-activated proton channel [31]. The M2 channel acidifies the interior of the virion during entry to aid in unpacking the genome [32]. For some strains of influenza virus, M2 also plays an important role in the secretory pathway where it raises the pH of the *trans*-Golgi to prevent the premature activation of the fusion protein [29], [33], [34]. Hepatitis C virus (HCV), like CoVs, assembles intracellularly and must navigate the secretory pathway for release. Interestingly, HCV encodes a proton selective viroporin, p7 [35]–[37]. While the exact role p7 is not fully understood, it is important in the assembly and release of HCV virions, and expression of p7 leads to the alkalinization of secretory organelles [37]–[39]. It is possible that HCV-p7 and CoV E have analogous roles during infection for altering the secretory pathway to promote the release of virions. Viroporins appear to play important roles in the assembly and trafficking of many viruses; understanding their exact role(s) is important as they represent good targets for therapeutic intervention via small molecule inhibitors.

While T16 in IBV E is required for disrupting the secretory pathway, it is not important for virus assembly as judged by VLP production. Our VLP results also suggest that disruption of the secretory pathway is not required for virus egress, since the T16A mutant produced the same level of VLPs as the wild-type E protein. However, the VLP assay does not allow measurement of infectivity, which was greatly reduced for particles released from cells infected with IBV carrying an E protein with a heterologous HD [21]. Another difference between infection and the VLP assay is that more particles are produced in a shorter time during infection, it is likely then that the stress on the secretory pathway is much more robust during infection. Thus, the VLP assay may not accurately reflect virion trafficking during infection. To measure the effect of T16 on virion trafficking, assays that measure both the amount, rate, and route of infectious particle trafficking are necessary. A future goal will be to analyze recombinant viruses carrying mutations at T16 with quantitative trafficking assays.

If CoV E is important for the release of infectious particles, why do some CoVs show only a modest reduction in infectivity when E is deleted [40]? Moreover, why do we only observe a measurable disruption in the secretory pathway with IBV E and not the E proteins from other CoVs? The answer to these questions may lie in the exact role(s) that the CoV E protein plays for each virus. While the E proteins from different CoVs share a similar domain structure, there is large variation in their primary sequence. Additionally, the requirement of CoV E for the production of infectious virus is not consistent between different CoVs. The E protein of the TGEV is essential for the production of infectious virus [18]. However, a version of MHV lacking the E gene can replicate, albeit at a greatly reduced titer [41]. Finally, a recombinant version of SARS-CoV with E deleted shows only a modest reduction in infectivity when passaged in cultured cell lines [40]. These results suggest that CoV E may have evolved to perform divergent functions in different CoVs. Somewhat surprisingly then, it was reported that the E protein from several different CoVs, including IBV E, could substitute for MHV E during infection [42]. Even more striking, when MHV ΔE was passaged, revertants were recovered with a partial duplication of the M gene (consisting of the N terminus and three transmembrane domains but lacking the C-terminal tail) that were able to largely compensate for the lack of E [43]. Taken together, these results show that at least some function(s) of the E protein are conserved among CoVs. However, the requirement for its function(s) may vary significantly due to the compensatory action of other viral proteins or differences in cell and tissue types infected. Of all the CoVs whose E proteins were tested here, IBV is the only one with an avian host. The requirements for assembly and release in avian species may be slightly different than in mammals. We tested whether the disruption of the secretory pathway caused by IBV E occurred in DF-1 chicken fibroblasts (cultured at 39°C), and found that the secretory pathway was disrupted similar to HeLa cells (unpublished data). Another potential difference is the cell type in which each virus replicates. Certainly the requirements for virus egress in different tissues could be an important factor. Another possibility is that the compartmental localization of the E proteins may vary in the absence of the other viral proteins and the impact of each CoV E on the secretory pathway could depend on the Golgi subcompartment in which it is localized. This possibility could be addressed by immunoelectron microscopy on cells expressing the various E proteins. There is a notable difference in the ion specificity and channel behavior among the different E protein channels in planar lipid bilayers [4], [5]. Unlike the other CoV E channels characterized, the IBV E channel demonstrated rectification, where ions are moved predominately in one direction [5]. Additionally, the IBV E channel is insensitive to the small molecule HMA, unlike the other CoV E proteins tested [5], [9]. If the ion specificity or activity varies between the CoV E proteins, it could certainly explain the differences in behavior reported here. The best way to study these differences would require electrophysiological measurements using patch clamp analysis on purified Golgi membranes. This approach would allow the direct measurement of the CoV E protein in its natural membrane with the proper post-translational modifications, but will be very technically challenging. One last point is that the sequences of the CoV E proteins are highly variable. Of note, IBV E is significantly larger and contains more polar residues in its HD than the other CoV E proteins (see Figure 3A). It will be important to determine how these differences relate to the function of the proteins. This could be addressed by determining how chimeric proteins affect the secretory pathway and virus replication.

Previous reports on CoV E protein topology have suggested that it may exist either as a transmembrane protein or as a membrane hairpin with both the N- and C-termini in the cytoplasm. The ability to adopt multiple membrane topologies could be a mechanism to increase the number of protein functions within the constrictions of genome size. Here, we generated mutant versions of IBV E that adopted either a membrane hairpin or transmembrane topology. We found that the transmembrane version of the protein behaved largely like IBV E, with the exception that it was unable to drive VLP production to the same degree. The membrane hairpin version of IBV E was unable to disrupt the secretory pathway or drive VLP production. These data suggest that IBV E largely functions as a transmembrane protein, with no apparent role for the membrane hairpin. However, such conclusions should be drawn with caution. While we determined that ssFLAG-IBV E behaved largely like ssIBV E, addition of the FLAG tag onto the N-terminus of IBV E could have any number of off-target effects, especially when considering the interaction of the E protein with M. We attempted to generate a membrane hairpin using several different strategies, including altering the charge distribution on either end of the HD, extending the N terminus with different tags, and shortening the C-terminus. Our only successful strategy was adding the FLAG tag onto the N-terminus. It should be noted that all reports of CoV E demonstrating that it adopts a membrane hairpin upon expression have been carried out using N-terminally tagged proteins [15], [16]. In fact the most recent data on the topology of SARS-CoV E using the untagged protein and antibodies directed to either terminus show that the predominant topology is N_exo_, C_cyto_ [13]. What remains unclear is if a membrane hairpin plays a role during infection. It is possible that a portion of the E protein adopts a membrane hairpin topology. We did observe a small difference between ssIBV E and IBV E when we quantified the signal from our selective permeabilization experiment. A small amount of CoV E in the membrane hairpin conformation could play a catalytic role during assembly, and while not necessarily required for assembly, it may increase the efficiency of assembly. This would explain why FLAG-IBV E could not support VLP production on its own. This idea could be addressed by developing infectious clones of IBV carrying the topology mutants of IBV E and examining particle production biochemically and by electron microscopy of infected cells. Also of interest is the mechanism for generation of multiple topologies. A transmembrane topology is likely generated through the canonical signal recognition particle pathway like other type III membrane proteins [23], but the generation of a hairpin could involve a different mechanism. One could speculate that a hairpin could be generated through post-translational insertion, possibly directly into the target membrane [44].

The IBV E protein is a multifunctional viral protein that plays a role in both the assembly and release of infectious virus. The exact mechanism by which the protein alters the secretory pathway to facilitate infectious particle release is still unknown, but may depend on a single amino acid in the HD. Identification of the mechanism will be a big step in understanding the interplay between the secretory pathway and CoV trafficking. Also of interest is how E protein function varies among CoVs and what underlies any difference(s). Understanding these questions will provide insight into both therapeutic approaches to CoV infection and increase our understanding of how CoVs use the host secretory pathway to their advantage.

## Materials and Methods

### Cell Culture and Transfection

HeLa cells were cultured in Dulbecco's Modified Eagle Medium (DMEM) (Invitrogen) with 10% Fetal Bovine Serum (FBS) (Atlanta Biologicals), and 0.1 mg/ml Normocin (InvivoGen) at 37°C under 5% CO_2_. Transient transfection of HeLa cells was performed using Fugene6 or XtremeGene 9 (Roche) according to the manufacturer's protocol. Experiments were performed 18–22 hours post transfection unless noted otherwise.

### Plasmids and Mutagenesis

The expression plasmids for IBV E, VSV G and IBV M have previously been described [12], [21], [45]. The sequence for IBV N was amplified by RT-PCR of RNA from IBV infected cells. The sequence was inserted into pcDNA3.1 using BamHI and EcoRI sites, and subcloned into pCAGGS using KpnI and XhoI. Mutations of the HD of IBV E were introduced via Quikchange (Stratagene) site directed mutagenesis. ssIBV E was generated by inserting a BglII site directly upstream of the start codon of IBV E using Quikchange mutagenesis. The vector was digested with EcoRI and BglII and synthetic oligonucleotides encoding the signal sequence of VSV G (MKCLLYLAFLFIGVNCRS) with flanking EcoRI and BlgII sites was ligated upstream of the start of IBV E to generate ssIBV E. The FLAG-IBV E construct was made in a similar way. A sequence encoding an initiation codon and the FLAG epitope (MDYKDDDDK) with flanking BglII sites was ligated directly upstream of the start codon of IBV E. ssFLAG E was generated by ligating the same FLAG epitope (MDYKDDDDK) into the ssIBV E construct after digestion with BglII. pCAGGS SARS E (Urbani) has been previously described [46]. Plasmids containing the coding sequences for MHV E (A59) and TGEV E (Purdue p115) were kindly provided by Paul Masters (Wadsworth Center, Albany, NY). The coding sequence of MHV E and TGEV E were PCR amplified and inserted into pCAGGS using EcoRI and KpnI or EcoRI and XhoI respectively. The CFP-KDEL expression vector was from clontech. The construct consists of a signal sequence followed by the cyan fluorescent protein and a KDEL ER retrieval sequence in the C-terminal tail of the protein. Golgin160-myc has been previously described [47].

### Antibodies

The following antibodies have been previously described: Rabbit and rat antibodies recognizing the C termini of IBV E, rabbit antibody recognizing the N terminal portion of IBV E [12], rabbit anti-IBV M used for immunoblotting [48], and the rabbit anti-VSV polyclonal antibody used for immunoprecipitation [49]. The rabbit anti-MHV E and rabbit anti-TGEV E used for immunofluorescence were kind gifts from Paul Masters, and have been previously described [42]. The rabbit anti-IBV N antibody was a kind gift from Ellen Collisson and has been previously described [50]. Mouse anti-GM130 was from BD Biosciences, rabbit anti-GFP was from Molecular Probes, mouse anti-FLAG M2 was from Sigma, and the monoclonal mouse anti-Myc antibody (clone 9E10) was from Roche Molecular Biochemicals. The Alexa Fluor 488 conjugated donkey anti-rabbit IgG, Alexa Fluor 488 conjugated donkey anti-mouse IgG, Alexa Fluor 568 conjugated donkey anti-rabbit IgG and Alexa Fluor 568 conjugated anti-mouse IgG were from Molecular Probes. The Texas Red conjugated donkey anti-rat was from Jackson ImmunoResearch Laboratories. The horseradish peroxidase conjugated donkey anti-rabbit antibody was from Amersham.

### Multiple Sequence Alignment

Multiple sequence alignment of CoV E proteins was carried out using ClustalW2 at the European Bioinformatics Institutes server [51]. The figure was generated using jalview version 2 [52]. GenBank accession numbers of the sequences used in the alignment are as follows: TGEV E (ABG89321), IBV E (CAC39117), SARS E (NP_828854.1), MHV E (ACO72886), FIPV E (AAY16378), HCoV HKU1 (YP_173240), PEDV (NP_598312), PHEV (YP_459955), Bovine CoV (NP_150081), HCoV OC43 (NP_937952), and HCoV 229E (NP_073554).

### Pulse-Chase Endo H assay

HeLa cells were transfected with pCAGGS VSV G (1 µg) along with either a control plasmid (0.5 µg pCAGGS IBV M) or a pCAGGS E construct (0.5 µg). Cells were incubated in cysteine-methionine free DMEM for 15 min, labeled with 50 µCi of Expre^35^S^35^S [^35^S]-methionine-cysteine (Perkin Elmer) in cysteine-methionine free DMEM for 20 min, and chased in normal growth medium. Prior to collection, labeled cells were washed with PBS. Samples were lysed in detergent solution with protease inhibitor cocktail and clarified at 20,000×g. SDS was added to 0.2% and the samples were pre-cleared with *Staphylococcus aureus* Pansorbin cells. Rabbit anti-VSV antibody was added to each sample and incubated for 20′. Immune complexes were collected with 20 µl of washed *Staphylococcus aureus* Pansorbin cells and washed two times in RIPA buffer (10 mM Tris [pH 7.4], 0.1% SDS, 1% deoxycholic acid, 1% NP40, 150 mM NaCl). Immune complexes were eluted in 1% SDS [pH 6.8] at 100°C and digested in 75 mM Na-citrate [pH 5.5] with 0.2 µl endo H (100 units) (New England Biolabs) at 37°C overnight. Concentrated sample buffer (200 mM Tris-HCl [pH 6.8], 8% SDS, 60% glycerol, 0.2% bromophenol blue) was added to each sample prior to separation on 10% SDS-PAGE. Labeled proteins were visualized by using a Molecular Imager FX phosphorimager (Bio-Rad) and quantified using Quantity One software (BioRad).

### Indirect Immunofluorescence Microscopy

HeLa cells plated on glass coverslips were processed for immunofluorescence 18–22 h after transfection. For assaying Golgi disruption in cells expressing IBV E or the HD mutants cells were fixed in 3% paraformaldehyde for 10 min. The fixative was quenched with PBS containing 10 mM glycine (PBS/Gly). The cells were permeabilized in 0.5% TX-100 for 3 min and washed in PBS/Gly. Cells were stained with rabbit anti-IBV E (1∶1000) and mouse anti-GM130 (1∶1000). Secondary antibodies were Alexa Fluor 488 conjugated anti-rabbit IgG (1∶1000), Alexa Fluor 568 conjugated anti-mouse IgG (1∶1000). DNA was stained prior to imaging with Hoechst 33285 (0.1 µg/ml). All images were collected using an Axioscop microscope (Zeiss) equipped for epifluorescence using an ORCA-03G charge-coupled-device camera (Hamamatsu, Japan). Data analysis was done using iVision software (BioVision Technologies) and Microsoft Excel. To determine if the Golgi complex was disrupted in cells expressing IBV E or its mutants, the staining for the Golgi complex (as judged by the GM130 staining) was outlined. The area encompassing the Golgi complex was measured for cells expressing IBV E, the HD mutants, or non-transfected cells. A normal Golgi was determined to be the average area of non-transfected cells +/−1.5 standard deviations. Cells with a staining area larger than this were scored as disrupted. The percent disrupted was calculated by dividing the number of cells scored as disrupted by the total number of cells measured.

### Selective Permeabilization of the Plasma Membrane

HeLa cells were transfected with CFP-KDEL (0.2 µg) and golgin160-Myc (1 µg) for control samples or with IBV E (0.5 µg), pCAGGS-ssIBVE (1.5 µg), pCAGGS-FLAG-IBV E (0.5 µg), pCAGGS ssFLAG-IBV E (0.5 µg), pCAGGS IBV E T16A (0.5 µg). For the Triton samples the protocol listed above was followed. For selective permeabilization of the plasma membrane, cells were washed with a cold KHM (20 mM HEPES [pH 7.4], 110 mM KOOCH_3_, 2 mM Mg(OOCH_3_)_2_) and kept on ice. The cells were permeabilized with 75 µg/ml digitonin (EM Sciences) for 10 min. The digitonin solution was removed and the cells were rinsed twice with cold KHM. The cells were moved to room temperature and fixed with 3% paraformaldehyde for 10 min. The control cells were incubated with mouse anti-Myc (1∶2) and rabbit anti-GFP (1∶500). The C-terminus of IBV E, ssIBV E, and FLAG-IBV E were detected using a C-terminal rat anti-IBV E antibody (1∶500). The N-terminus of IBV E and ssIBV E was detected using a rabbit anti N-terminal IBV E antibody (1∶100). The N-terminus of FLAG-IBV E was detected using a mouse anti-FLAG antibody (1∶500). Secondary antibodies were The Alexa Fluor 488 conjugated donkey anti-rabbit IgG (1∶1000), Alexa Fluor 568 conjugated anti-mouse IgG (1∶1000), and Texas Red conjugated donkey anti-rat (1∶500). DNA was stained prior to imaging with Hoechst 33285 (0.1 µg/ml).

For quantitation, images of equal exposure time were taken of both the Triton X-100 and digitonin samples for each antibody. To obtain the staining intensity, an initial background measurement was obtained on the C-terminal staining by drawing a region of interest (ROI) around an untransfected cell and measuring the fluorescence intensity. The exact same ROI and measurement was then made on the corresponding N-terminal image. Next, an ROI was drawn around a cell showing C-terminal staining and the mean fluorescence intensity was measured. Again, the exact same ROI was overlaid onto the corresponding N-terminal image and the mean fluorescence intensity was measured. The ratio of N- to C-terminal staining was calculated by first subtracting the background from each measurement, and then dividing the N-terminal value by the C-terminal value. For the data shown, the final ratios were normalized so that the signal ratio in the Triton X-100 samples was equal to 1.

### Virus Like Particle Isolation

HeLa cells were plated in 6 cm dishes and transfected with a combination of plasmids encoding IBV M (2 µg), IBV N (1.5 µg), IBV E (0.1 µg), ssIBV E (0.4 µg), FLAG-IBV E (0.2 µg), ssFLAG IBV E (0.2 µg), T16A (0.1 µg), T16S (0.1 µg), T16N (0.1 µg) and T16N (0.1 µg). Samples were prepared 42–48 hours post transfection. The medium was clarified via centrifugation at 4500×g for 20 min. The supernatant was loaded onto a 20% sucrose cushion and centrifuged at 234,000×g in a TLA-110 rotor for 60 min. The supernatant was discarded and the pellet containing the VLPs was resuspended in 1× glycoprotein denaturation buffer (New England Biolabs) containing 100-fold concentrated protease inhibitor cocktail (Sigma). To collect the cell fraction, dishes were washed with cold PBS. The cells were scraped off the dish in 1 ml PBS and pelleted at 4000×g for 2 min. The pellet was resuspended in detergent solution and insoluble material was pelleted at 20,000×g for 1 min. 10× glycoprotein denaturation buffer was added to 1×. Both the VLP and cell fractions were heated at 100°C for 1 min. Both samples were digested with PNGase F (New England Biolabs) according to the manufacturer's protocol. After digestion concentrated sample buffer was added to a final concentration of 50 mM Tris [pH 6.8], 2% SDS, 0.05% bromophenol blue, 15% glycerol. Samples were separated on 15% PAGE gels (10% of cell fraction, 100% of VLP fraction) and transferred to polyvinylidene fluoride Immobilon membranes (Millipore). Proteins were detected using rabbit anti-IBV N (1∶10,000), rabbit anti-IBV M (1∶5000) and rabbit anti-IBV E (1∶10,000) primary antibodies and horseradish peroxidase conjugated donkey anti-rabbit IgG (1∶10,000) secondary antibody. After incubation in secondary antibody, the membrane was incubated with HyGlo Quick Spray chemiluminescent detection reagent (Denville Scientific Inc.). Images were collected using a Versa Doc model 5000 (Bio-Rad) and Quantity One software.

### Cycloheximide Chase to estimate half-lives of CoV E proteins

HeLa cells were treated with 100 µg/ml cycloheximide (Sigma) diluted into culture media at 18 hours-post transfection. Cells were fixed and prepared for immunofluorescence as described above at 0, 3, and 6 hrs after cycloheximide treatment. Images were collected from each time point at the same exposure time, and the mean fluorescence intensity was determined for cells expressing the E protein. The half-lives of each E protein were calculated by plotting the signal intensity versus time on a semi-log graph.

## Supporting Information

Figure S1 Expression of other CoV E proteins does not disrupt Golgi complex morphology. Indirect immunofluorescence microscopy on cells expressing TGEV E, MHV E, or SARS-CoV E. The E protein is shown in green, GM130 is shown in red, and nuclei are shown in blue. Scale bars, 10 µm.
(TIF)

Figure S2 IBV E T16A has the same topology as IBV E. Selective permeabilization was carried out on cells expressing IBV E T16A. The N- and C-termini were detected using antibodies specific to each terminus. The histogram shows quantification of topology as a ratio of the N-terminus to C-terminus fluorescence signal (see Material and Methods). The data are normalized to the ratio from the Triton X-100 permeabilized samples. Data are from at least 2 independent experiments with N≥16 for each condition. Error bars represent +/− SEM, and the asterisk denotes a significant difference between the Triton X-100 and digitonin signal by Student's *t*-test (p≤2.5×10^−7^).
(TIF)

Figure S3 ssFLAG-IBV E behaves similarly to ssIBV E. (A) Indirect immunofluorescence microscopy on cells expressing ssFLAG-IBV E. The E protein is shown in green, GM130 is shown in red, and nuclei are shown in blue. Scale bar, 10 µm. (B) Selective permeabilization was carried out on cells expressing ssFLAG-IBV E. The N- terminus was detected using an anti-FLAG antibody and the C-terminus was detected using a Rat anti-IBV E antibody. The histogram shows quantification of topology as a ratio of the N-terminus to C-terminus fluorescence signal (see Material and Methods). The data are normalized to the ratio from the Triton X-100 permeabilized samples. Data are from at least 2 independent experiments with N≥22 for each condition. Error bars represent +/− SEM, and the asterisk denotes a significant difference between the Triton X-100 and digitonin signal by Student's *t*-test (p≤1×10^−8^). (C) Quantification of Golgi complex disruption in HeLa cells expressing ssFLAG-IBV E (see Figure 2 for description of quantification). Data are from 2 independent experiments with N≥37 for each condition. Error bars represent +/−SEM. (D) The graph shows the quantification of VSV G pulse-chase coupled with endo H digestion as described in Figure 1. ssFLAG-IBV E dramatically affects cargo trafficking. Data are from 2 independent experiments. Error bars represent +/− SEM. (E) A VLP assay was performed and quantified as described in Figure 4 for ssFLAG-IBV E. ssFLAG-IBV E was compromised in the production of VLPs compared to IBV E. Data are from at least five independent experiments. Error bars represent +/− SEM, and the asterisk denotes a significant decrease in VLP level compared to IBV E by Student's *t*-test (p<1.4×10^−4^).
(TIF)
