# Supplementary figures and images for: A Single Polar Residue and Distinct Membrane Topologies Impact the Function of the Infectious Bronchitis Coronavirus E Protein
Source: PLoS Pathog. 2012 May 3;8(5):e1002674. doi: 10.1371/journal.ppat.1002674 (PMC3343006; doi:10.1371/journal.ppat.1002674)

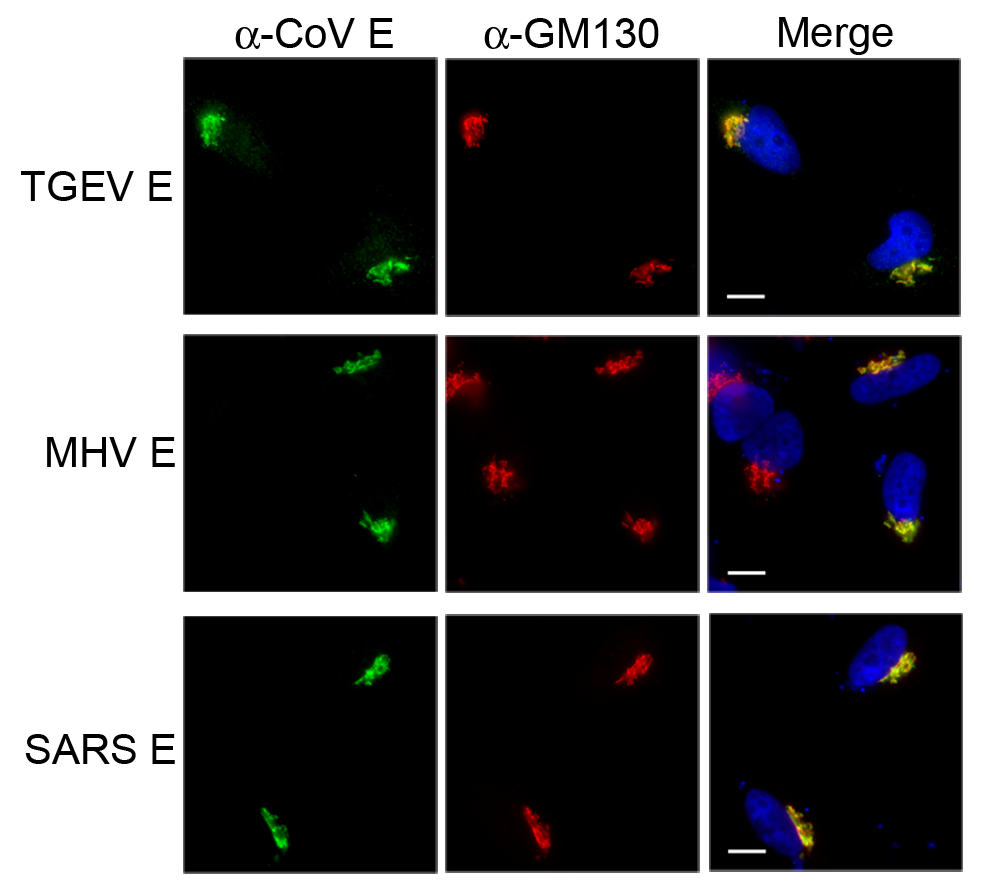

Supplement: Figure S1 — Expression of other CoV E proteins does not disrupt Golgi complex morphology. Indirect immunofluorescence microscopy on cells expressing TGEV E, MHV E, or SARS-CoV E. The E protein is shown in green, GM130 is shown in red, and nuclei are shown in blue. Scale bars, 10 µm. (TIF) [file ppat.1002674.s001.tif]

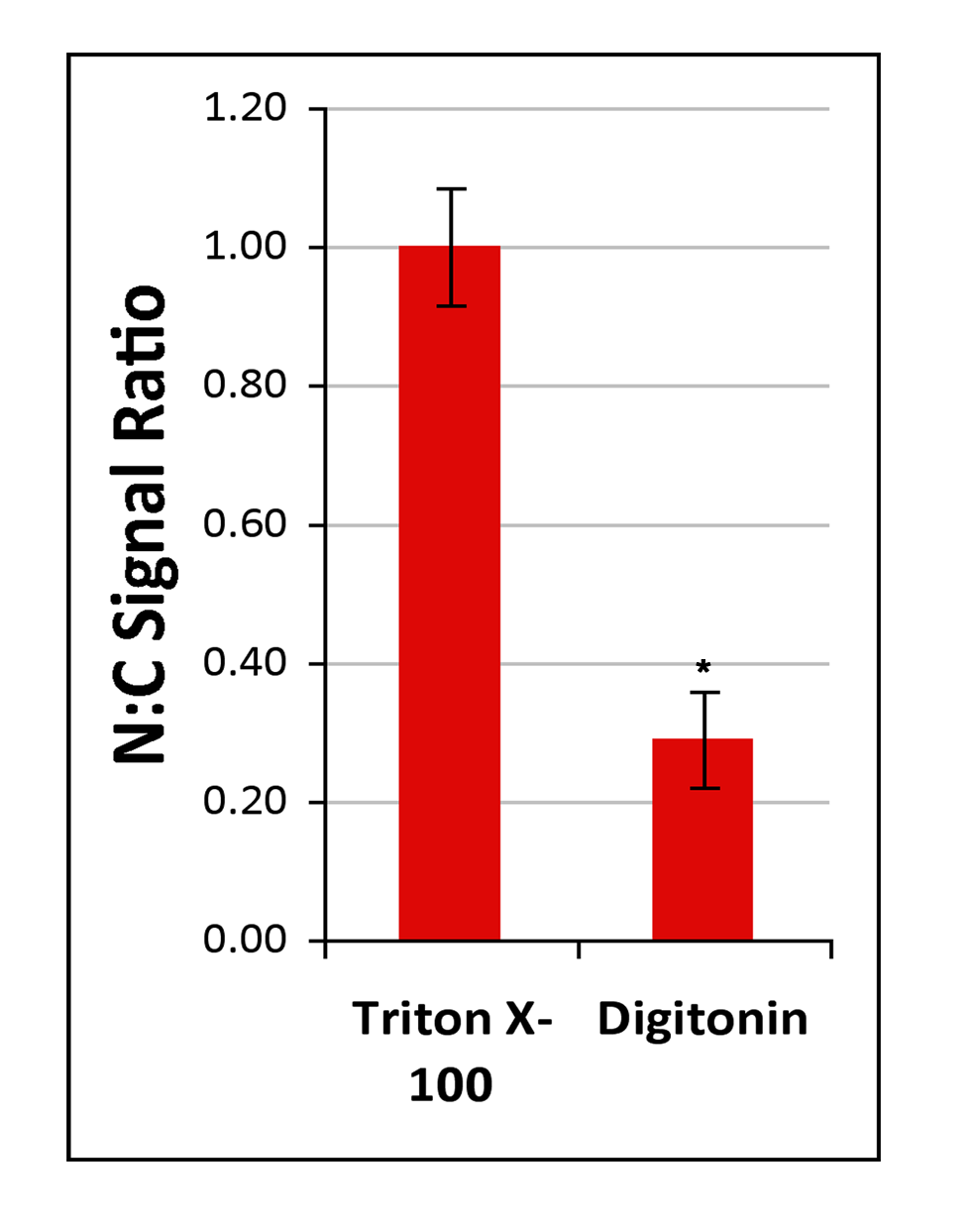

Supplement: Figure S2 — IBV E T16A has the same topology as IBV E. Selective permeabilization was carried out on cells expressing IBV E T16A. The N- and C-termini were detected using antibodies specific to each terminus. The histogram shows quantification of topology as a ratio of the N-terminus to C-terminus fluorescence signal (see Material and Methods). The data are normalized to the ratio from the Triton X-100 permeabilized samples. Data are from at least 2 independent experiments with N≥16 for each condition. Error bars represent +/− SEM, and the asterisk denotes a significant difference between the Triton X-100 and digitonin signal by Student's t-test (p≤2.5×10^−7^). (TIF) [file ppat.1002674.s002.tif]

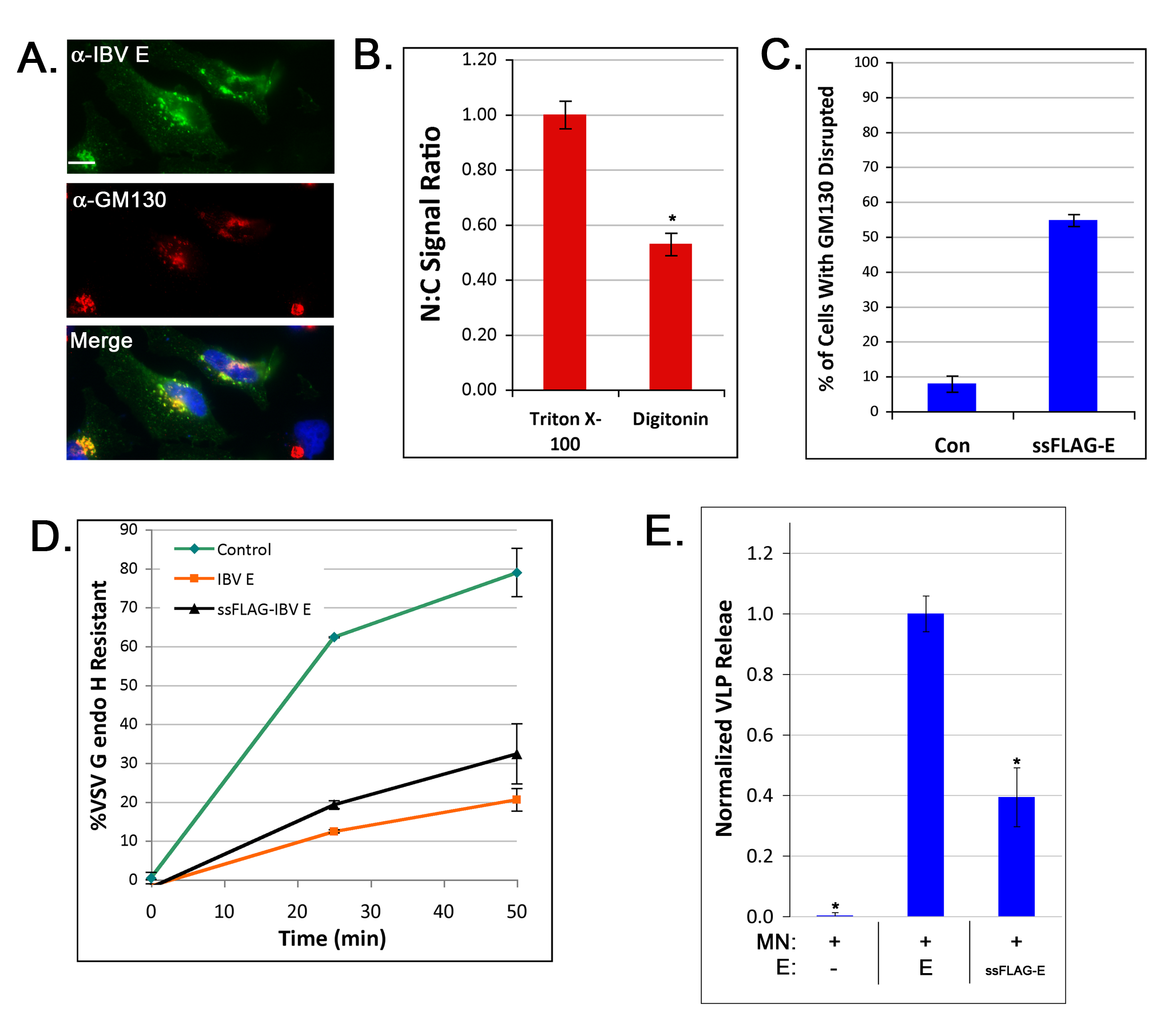

Supplement: Figure S3 — ssFLAG-IBV E behaves similarly to ssIBV E. (A) Indirect immunofluorescence microscopy on cells expressing ssFLAG-IBV E. The E protein is shown in green, GM130 is shown in red, and nuclei are shown in blue. Scale bar, 10 µm. (B) Selective permeabilization was carried out on cells expressing ssFLAG-IBV E. The N- terminus was detected using an anti-FLAG antibody and the C-terminus was detected using a Rat anti-IBV E antibody. The histogram shows quantification of topology as a ratio of the N-terminus to C-terminus fluorescence signal (see Material and Methods). The data are normalized to the ratio from the Triton X-100 permeabilized samples. Data are from at least 2 independent experiments with N≥22 for each condition. Error bars represent +/− SEM, and the asterisk denotes a significant difference between the Triton X-100 and digitonin signal by Student's t-test (p≤1×10^−8^). (C) Quantification of Golgi complex disruption in HeLa cells expressing ssFLAG-IBV E (see Figure 2 for description of quantification). Data are from 2 independent experiments with N≥37 for each condition. Error bars represent +/−SEM. (D) The graph shows the quantification of VSV G pulse-chase coupled with endo H digestion as described in Figure 1. ssFLAG-IBV E dramatically affects cargo trafficking. Data are from 2 independent experiments. Error bars represent +/− SEM. (E) A VLP assay was performed and quantified as described in Figure 4 for ssFLAG-IBV E. ssFLAG-IBV E was compromised in the production of VLPs compared to IBV E. Data are from at least five independent experiments. Error bars represent +/− SEM, and the asterisk denotes a significant decrease in VLP level compared to IBV E by Student's t-test (p<1.4×10^−4^). (TIF) [file ppat.1002674.s003.tif]
